# Identification of *Aspergillus nomius* in Bees Visiting Brazil Nut Flowers

**DOI:** 10.1264/jsme2.ME14146

**Published:** 2015-06-09

**Authors:** Fernanda Pelisson Massi, Rafael Elias Silva Penha, Marcelo Casimiro Cavalcante, Helena Paula Viaro, Josué José da Silva, Larissa de Souza Ferranti, Maria Helena Pelegrinelli Fungaro

**Affiliations:** 1 Centro de Ciências Biológicas, Departamento de Biologia Geral, Universidade Estadual de Londrina, P.O. Box 6001, 86051–990, Londrina, Paraná, Brazil; 2 Departamento de Ciência Animal, Universidade Federal do Ceará, 60021–970, Fortaleza, Ceará, Brazil

**Keywords:** aflatoxin, *Aspergillus* section *Flavi*, molecular detection, pollinators of *Bertholletia excelsa*

## Abstract

We designed a primer pair (BtubNomF/BtubNomR) specifically for amplifying *Aspergillus nomius* DNA. *In vitro* assays confirmed BtubNomF/BtubNomR specificity, corroborating its usefulness in detecting and identifying *A. nomius*. We then investigated the occurrence of *A. nomius* in floral visitors of *Bertholletia excelsa* trees by means of PCR, and *A. nomius* was detected in the following bees: *Xylocopa frontalis*, *Bombus transversalis*, *Centris denudans*, *C. ferruginea*, and *Epicharis flava*. The presence of *A. nomius* in bees visiting Brazil nuts opens up new avenues for obtaining novel insights into the process whereby Brazil nuts are contaminated by aflatoxin-producing fungi.

The Brazil nut (*Bertholletia excelsa*) is a South American tree native to the Amazonian rainforest. It can reach a height of 45 meters. Flowering begins at the end of the rainy season in September and extends to February (5). The flower is large and zygomorphic with two or three sepals and six yellowish petals. It incorporates a curled hood formed by congruent staminodes (ligule) which, with the petals, forms a chamber containing the stamens, stigma, and nectaries. The size and robustness of the hood mean that pollination is restricted to medium and large-sized bees strong enough to uncurl it, predominantly *Eulaema mocsaryi* and *Xylocopa frontalis* (6).

The fruit is an extremely hard spherical capsule of a ligneous mesocarp. Inside the fruit, the seeds (or nuts) have a ligneous rough shell. The fruit remains on the tree for 15 months. When mature, it falls to the ground where it interacts with the soil for days or weeks before it is harvested.

The occurrence of aflatoxins in Brazil nuts is one of the main obstacles to their commercialization. Although soil has been systematically identified as the dominant source of fungal contamination (1, 3), the incidence of aflatoxigenic fungi in nuts collected directly from the trees was previously reported to be high (2).

Among the aflatoxigenic fungi, *Aspergillus nomius*, belonging to section *Flavi*, has been identified as the main culprit for the presence of aflatoxins in Brazil nuts (4, 8, 10–12). The taxonomy of *Aspergillus* section *Flavi* is complex, and it can be very difficult, and even impossible to identify some species morphologically.

In the present study, strains of *Aspergillus* section *Flavi* were analyzed using β-tubulin gene sequences in order to develop specific primers for the detection of *A. nomius*. The primer pair provided herein was used to verify the possible incidence of *A. nomius* in visitor bees of Brazil nuts, thereby opening up new possibilities for deepening our knowledge of the process whereby Brazil nuts are contaminated by aflatoxin-producing fungi.

In order to develop specific primers for detecting *A. nomius*, the β-tubulin gene sequences from the *A. nomius* clade (*A. pseudonomius*, *A. bombycis* and *A. nomius*) were downloaded from the GenBank database and aligned using BioEdit v.7.0.5.3 software (9). Nineteen of the *A. nomius* sequences deposited (NRRL 3353; NRRL 6552, NRRL 26455; NRRL 26886; NRRL 26885; NRRL 26883; NRRL 5919; 823/07; NRRL 26454; NRRL 26452; NRRL 26450; NRRL 26451; NRRL 26881; NRRL 26879; NRRL 6343; NRRL 26878; NRRL 26882; NRRL 26880; NRRL 26884) were interpreted in our study as belonging to *A. pseudonomius*, as recommended in a recent study (10). The sequence variations of all 50 taxa belonging to the *A. nomius* clade, available in the GenBank database (accessed June 14, 2014), allowed us to define eight distinct haplotypes based on the *benA* locus. These eight haplotypes, representing strains around the world, were used to design the primer pair denoted as BtubNomF and BtubNomR (5′ AGC AGA AAC ATG AGC TCG GAT A 3′ and 5′ TTC CCG TCA GAC CCA TCC A 3′) to specifically amplify *A. nomius* DNA (Fig. 1). In order to predict specificity to *A. nomius* strains, this primer combination was tested *in silico* against all *Aspergillus* section *Flavi* species. We found that both primers were 100% identical to all *A. nomius* strains, but, when combined, were not 100% identical to any of the other strains of the species belonging to the section *Flavi*.

All *Aspergillus* section *Flavi* strains available in our laboratory (*A. flavus* (*n*=10), *A. arachidicola* (*n*=2), *A. tamarii* (*n*=2), *A. pseudotamarii* (*n*=2), *A. caelatus* (*n*=2), *A. pseudocaelatus* (*n*=2), *A. bertholletius* (*n*=5), *A. pseudonomius* (*n*=6), *A. bombycis* (*n*=2) and *A. nomius* (*n*=34)) were used to assess the specificity of the primer pair designed to detect *A. nomius*. The PCR reaction was carried out in a total reaction volume of 25 μL containing 1×PCR buffer, 2.0 mM of MgCl_2_, 0.2 mM of dNTP mixture, 0.4 μM of each primer, 1 U of *Taq* DNA polymerase (Invitrogen, Life Technologies), and 10 ng of DNA template. The amplification program consisted of one cycle at 95°C for 5 min, then 34 cycles at 95°C for 30 s, 65°C for 30 s, and 72°C for 30 s, and a final extension step at 72°C for 4 min. Under these PCR conditions, the primer pair BtubNomF/BtubNomR provided an amplicon of 185 bp for all *A. nomius* strains, but did not produce PCR products of the correct size from any of the other *Aspergillus* section *Flavi* species available in our collection, indicating that it may be used to identify *A. nomius* in cultures (Fig. 2).

Since *A. nomius* is often associated with insects (14), we decided to investigate, by means of PCR, the presence of *A. nomius* in bees visiting *B. excelsa*. Floral visitors were captured in a Brazil nut plantation situated at kilometer 215 on the Manaus-Itacoatiara road in the Brazilian state of Amazonas (3°2′S 58°45′W). This area is part of the Aruanã farm, extending over 12,000 ha, including 3,600 ha planted with Brazil nut trees, spaced 20 m apart. It is the largest Brazil nut plantation in the world, with approximately 1,300,000 trees. Bees visiting flowers were collected using entomological nets. They were then killed in an ethyl acetate killing jar, and individual species were identified using morphological characteristics. To extract bee DNA, we removed the abdomens of *Xylocopa frontalis* (*n*=8), *Bombus transversalis* (*n*=1), *Centris denudans* (*n*=2), *C. ferruginea* (*n*=1), and *Epicharis flava* (*n*=1). The remaining materials were ground with a mortar and pestle under liquid nitrogen and homogenized with extraction buffer (50 mM Tris-HCl, pH 8.0; 1% SDS; 0.75 M NaCl; 20 mM EDTA). Proteinase K solution (100 mg mL^−1^) was then added to the mixture. Samples were incubated at 64°C for 2 h and the resulting solution was deproteinized with phenol-chloroform standard protocol. Isopropanol was used to precipitate nucleic acids, which were then resuspended in 50 mL TE (10 mM Tris-HCl, pH 8.0; NC indicates negative control. Only *A. nomius* strains showed the 185 bp specific amplicon. Aspergillus nomius *in Bees Visiting Brazil Nuts* 275 1 mM EDTA). A portion of the cytochrome b gene (13) from extracted DNA was amplified using PCR with the primers CB-J-10933F (5′ TAT GTA CTA CCA TGA GGA CAA ATA TC 3′) and CB-N-11367R (5′ ATT ACA CCT CCT AAT TTA TTA GGA AT 3′). Amplified DNA fragments were observed in the DNA of all bees with the expected lengths by agarose gel electrophoresis.

In order to investigate the presence of fungal species in the bees collected, standard PCR was run on the DNA of all bees using the β-tubulin universal primers Bt2a/Bt2b described previously (7). Amplification products showing the presence of fungus (590 bp) were obtained from seven samples of *X. frontalis*, one sample of *B. transversalis*, two samples of *C. denudans*, one sample of *C. ferruginea*, and one sample of *E. flava*. The PCR products generated using the Bt2a/Bt2b primers were purified with the Wizard^®^ SV Gel and PCR Clean-Up System (Promega) diluted 100×. We then used 2 μL as a template in a NESTED-PCR reaction with the BtubNomF/BtubNomR primer pair, specifically designed to detect *A. nomius*. The PCR conditions were as described for testing primer specificity. By means of NESTED-PCR results, the *A. nomius* amplicon (185 bp) was detected in three samples of *X. frontalis*, and one sample each of *B. transversalis*, *C. denudans*, *C. ferruginea*, and *E. flava*. All NESTED-PCR products were sequenced and the BLASTn results confirmed the presence of *A. nomius*.

In order to confirm the effectiveness of NESTED-PCR for detecting *A. nomius* in bee visitors, two bulks of amplicons generated using the Bt2a/Bt2b (590 bp in size) were performed. The first bulk (A) included all seven PCR products that were positive for *A. nomius* while the second bulk (B) included all five PCR products that were negative for *A. nomius*. Both bulks were cloned separately into a sequencing vector (TOPO^®^ TA Cloning^®^ Kit; Invitrogen). The *E. coli* recombinant colonies (31 from bulk A and 21 from bulk B) were selected for insert sequencing. The inserts were sequenced for both orientations using the BigDye^®^ Terminator v 3.1 Cycle Sequencing kit (Applied Biosystems) and an ABI 3500XL Genetic Analyzer (Applied Biosystems), following the manufacturer’s instructions. Sequence identity verified using the NCBI BLASTn engine (http://blast.st-va.ncbi.nlm.nih.gov/Blast.cgi) corroborated the presence of *A. nomius* and *A. niger* in bulk A, and the presence of *Penicillium ochrochloron*, *Acremonium* sp., *Cyphellophora europaea*, *Phialophora europaea*, and *Discula* sp. in bulk B.

In conclusion, we succeeded in demonstrating for the first time the presence of *A. nomius* in bees visiting Brazil nuts, and the applicability of the BtubNomF/BtubNomR primer pair to its rapid diagnosis. We also obtained some new insights into the process of Brazil nut contamination by aflatoxin-producing fungi.

**Fig. 1:**
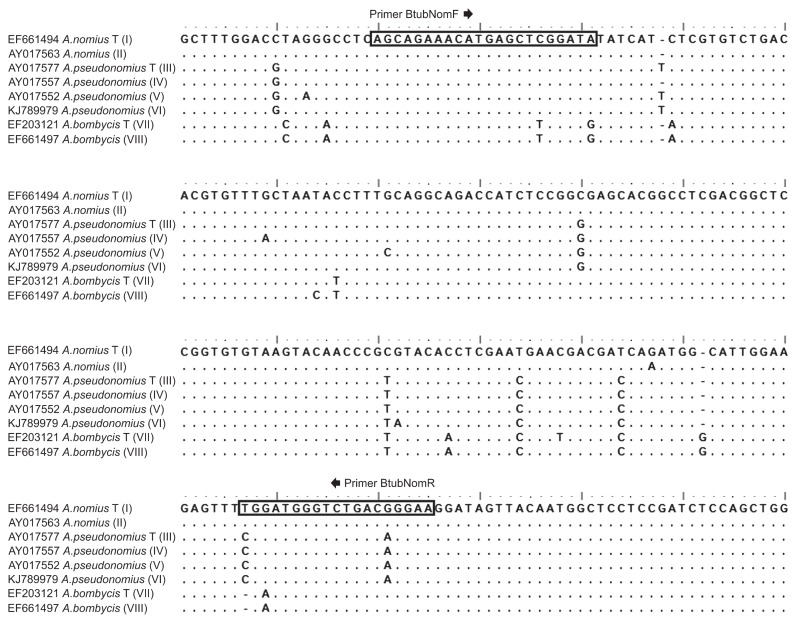
Primer pair designed specifically for amplifying *A. nomius* strains.

**Fig. 2:**
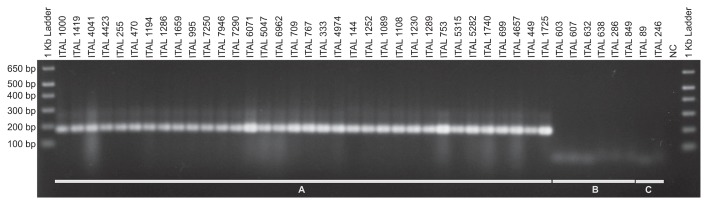
Results of PCR using the BtubNomF/BtubNomR primer pair to amplify *A. nomius* (A), *A. pseudonomius* (B), and *A. bombycis* (C) strains.
